# Unknown Renal Impairment: A Rare Case of Inhaled Tobramycin Induced Acute Kidney Injury in a Cystic Fibrosis Patient

**DOI:** 10.3390/antibiotics10040424

**Published:** 2021-04-12

**Authors:** Tyler Miller, Cristina Pastuch, Lisa Garavaglia, Kelley Gannon, Anthony Parravani

**Affiliations:** 1 Department of Medicine, West Virginia University, Morgantown, WV 26506, USA; 2 Department of Pediatrics and Medicine, West Virginia University, Morgantown, WV 26506, USA; cpastuch@hsc.wvu.edu; 3 Adult Cystic Fibrosis, Mountain State Cystic Fibrosis Center, West Virginia University, Morgantown, WV 26506, USA; 4 Department of Pharmaceutical Services, West Virginia University, Morgantown, WV 26506, USA; biondol@wvumedicine.org; 5 Section of General Internal Medicine, Department of Medicine, West Virginia University, Morgantown, WV 26506, USA; kgannon@hsc.wvu.edu; 6 Section of Nephrology, Department of Medicine, West Virginia University, Morgantown, WV 26506, USA; aparravani@hsc.wvu.edu

**Keywords:** cystic fibrosis (CF), tobramycin inhalation solution (TIS), tobramycin serum levels, acute kidney injury (AKI), chronic kidney disease (CKD)

## Abstract

Acute kidney injury is a reversible medical condition commonly caused by nephrotoxic agents. The infrequency that a nebulized medication elicits a renal insult presents a rare diagnostic challenge. Within this case, we report a 57-year-old cystic fibrosis patient with chronic kidney disease (CKD) Stage G3b (baseline 1.5–1.6 mg/dL) who developed an acute kidney injury (AKI) with a serum creatinine elevation to 4.08 mg/dL and associated worsening vestibular dysfunction related to twice-daily nebulized tobramycin inhalation solution (TIS). The patient was found to have a tobramycin serum level of 4.2 μg/mL 2.5 h after TIS dosing, with elevation remaining present at 1.1 μg/mL 24 h after discontinuation of therapy. Laboratory values at one month continued to show elevated creatinine levels at 2.1 mg/dL, suggesting progression of his baseline CKD. This case supports the benefit of obtaining tobramycin serum levels and vestibular/audiology function testing when evaluating patients on chronic nebulized TIS who present with acute or chronic renal dysfunction. From these serum levels, adjustments to daily dosing, regular monitoring of tobramycin serum levels, or discontinuation of treatment should be made to prevent permanent renal damage in patients with CKD. Calculated Naranjo ADR Probability Scale: 9; Definite.

## 1. Introduction

Acute kidney injury (AKI) is a reversible medical condition leading to an increase in serum creatinine and reduction in urinary output commonly caused by pre-renal (reduced blood flow secondary to hypotension, dehydration, or hemorrhage), renal (intrinsic insults such as glomerulonephritis, acute tubular necrosis, or nephrotoxic medications), or post-renal (renal calculi or lower urinary tract obstructions) causes. The presence of comorbid risk factors such as chronic kidney disease (CKD), congestive heart failure, diabetes, older age, sepsis, hypotension, or chronic medication use (non-steroidal anti-inflammatory drugs, angiotensin-converting enzyme inhibitors, angiotensin II receptor blockers, proton pump inhibitors, antibiotics, or chemotherapy agents) can place a patient at an increased risk for developing an AKI [1]. Traditional evaluation includes a thorough history, basic metabolic panel, urine electrolytes, urine analysis, and renal ultrasound. The goals of treatment are to identify and remove the insulting factor and treat with intravenous (IV) fluids to restore renal perfusion and prevent permanent damage [2]. If the diagnosis is delayed, progression to chronic kidney disease (CKD) can occur, which may necessitate long-term hemodialysis or renal transplant [3].

The prevalence of AKI and subsequent progression to CKD may be increased in patients with cystic fibrosis (CF). Cystic fibrosis is a genetic disease that affects more than 70,000 people worldwide by causing mutations within the cystic fibrosis transmembrane conductance regulator (CFTR) gene. This mutation effects chloride channels’ production of mucus and sweat, leading to thickened secretions. This increased mucus commonly leads to trapping of bacteria with associated colonization and long-term respiratory infections and inflammation. The disease also causes pancreatic insufficiency with associated poor growth, nutritional deficiencies, malabsorption, and often male infertility [4]. Risk factors for progression to CKD within a CF patient include older age, cystic fibrosis-related diabetes (CFRD), intermittent frequent IV aminoglycoside usage, and prolonged use of immunosuppressant therapy (tacrolimus, cyclosporine, azathioprine, and glucocorticoids) following solid organ transplantation [5,6]. The use of IV aminoglycosides with a second beta-lactam anti-pseudomonal antibiotic is commonly part of the standard treatment of a CF-pulmonary exacerbation. It has been shown that the number of pulmonary exacerbations and required hospitalizations a CF patient experiences increases with age and decline in FEV1 [7]. Over a lifetime, the cumulative doses of IV aminoglycosides may be substantial for an adult with CF and can potentiate the risk for CKD progression.

Protocols for CF aminoglycoside dosing is higher than standard protocols for the non-CF patient due to increased renal clearance [8]. This combination of higher dosing and frequent IV aminoglycoside usage places patients with CF at increased risk for CKD over their lifetime. Aminoglycoside antibiotics exhibit concentration-dependent bactericidal activity against susceptible organisms. Thus, the higher the serum concentration that is achieved, the more rapid the bactericidal activity. To maximize efficacy and minimize toxicity such as AKI, once-daily dosing (ODD) of IV aminoglycoside antibiotics has been determined a standard of care [9]. Current prophylactic strategies using tobramycin inhalation solution (TIS) for suppressive therapy has been shown to have greater airway penetration and higher antibiotic sputum levels than traditional IV routes [10,11]. TIS has been a critical advancement within CF treatment as it has been shown to reduce chronic infections, prevent exacerbations, improves lung function, and patient quality of life; it currently serves as a common first-line treatment for *Pseudomonas aeruginosa* [12]. Studies report sputum concentration of the drug is at least 25 times the minimal inhibitory concentration, with a ratio of median serum concentration to median sputum concentration of 0.01, making it an ideal method of administration [11].

Nebulized TIS common side effects include cough, shortness of breath, dizziness, and changes in voice. Limited research exists comparing the systemic involvement of nebulized TIS on acute and long-term renal impairment and vestibular dysfunction [9,13,14]. The infrequency that a nebulized medication can elicit a renal insult and present with detectable antibiotic serum levels presents a rare diagnostic challenge; reported cases are few and measurement of tobramycin levels are not standard practice. Within this case, we report a 57-year-old cystic fibrosis patient who developed an acute kidney injury on chronic kidney disease with associated worsening vestibular dysfunction related to twice-daily nebulized TIS.

## 2. Case Presentation

A 57-year-old male with cystic fibrosis (CF) with mutations F508del/c.3718-3T>G was admitted with fever, back pain, mild balance instability, hearing reduction, and loss of visual focus during movement. A concern for acute kidney injury on chronic kidney disease (Stage G3b) was noted after a review of routine annual laboratory evaluation. During the five days before admission, he also described progressive dynamic visual stabilization deficits with vestibular dysfunction.

His past medical history was significant for pancreatic insufficiency, chronic *Pseudomonas aeruginosa* pulmonary infection, CKD Stage G3b (serum creatinine 1.5–1.6 mg/dL), internal hemorrhoids, iron deficiency anemia, and previous neuronitis. The patient had no history of previous multidrug resistant pulmonary infections but had grown methicillin sensitive *Staphylococcus aureus* (MSSA) in prior sputum cultures, with last exacerbation in 2018. Medications included nebulized albuterol with hypertonic saline, dornase-alpha, azithromycin, pancreatic enzyme replacement therapy (PERT), anti-Pseudomonal suppressive inhalation continuous alternating therapy (CAT) with aztreonam inhalation solution (AIS) and tobramycin inhalation solution (TIS), and elexacaftor/tezacaftor/ivacaftor (Elexa/Tez/Iva) highly effective modulator therapy. He was currently on day 25 of 28 days of TIS.

On admission, the patient denied changes in urinary output, dysuria, hematuria, cough, shortness-of-breath, or sputum production. He felt “overall well and his lung health has improved since starting Elexa/Tez/Iva one year ago” with improved overall endurance. He denied any recent or chronic NSAID use, IV antibiotics, dehydration, or recent changes to daily medications.

Family history revealed cystic fibrosis and CHD post double lung transplant in a cousin and colon cancer in his father. The patient denied any tobacco, alcohol, or drug usage. Physical examination was normal except for bright red blood per rectum; he had clear lung fields, no edema or anasarca, and non-tender abdominal and flank examination. See Table 1 for laboratory values on admission.

Urine electrolytes were obtained to calculate FENa (fractional excretion sodium) and FEUrea (fractional excretion urea) in order to classify AKI. Results showed intrinsic renal cause suggesting glomerulonephritis, acute tubular necrosis, or nephrotoxic medications as the source for insult. Renal ultrasound showed a benign 8 mm non-obstructive right renal lower pole calculus without hydronephrosis present, a normal left kidney without calculus or hydronephrosis, an enlarged prostate, and a normal-sized bladder without debris or focal wall thickening.

The patient was continued on all medications including the TIS 300 mg twice a day, with the addition of intravenous (IV) hydration. On hospital day 3, a tobramycin level obtained 2.5 h after the nebulized dose was 4.2 μg/mL; 5.5 h later the level was 3.6 μg/mL. The TIS and Elexa/Tez/Iva were discontinued. A tobramycin level drawn 24 h after the last inhaled dose was 1.1 μg/mL.

Despite IV hydration and discontinuation of the TIS, the serum creatinine and potassium remained elevated. IV fluids were later discontinued and sodium-zirkonium cyclosilicate, sevelamer carbonate, sodium bicarbonate, and a low potassium diet were initiated. Urine sodium, urine osmolality, and serum osmolality were obtained after discontinuation of IV fluids suggesting continued intrinsic insult. Repeat FENa and FEUrea was not recalculated after IV fluid completion on day 7. His kidney function did not completely return to his chronic baseline. The patient was discharged on day 11 once serum creatinine stabilized. The patient did not require hemodialysis or renal biopsy due to plateau of serum creatinine. See Table 1 for all the laboratory values. Laboratory values at 1 month (day 41) continued to show elevated creatinine levels, noting possible CKD progression. Elexa/Tez/Iva and inhaled aztreonam were resumed at his cystic fibrosis follow-up appointment. The nebulized TIS was never resumed.

## 3. Discussion

This case presented a rare diagnostic challenge of acute kidney injury on chronic kidney disease (Stage G3b) in a patient with cystic fibrosis denying recent changes in medication, dosing, hydration, or new urinary retention. The patient additionally denied recent pulmonary exacerbations requiring IV antibiotics or steroids. His last noted exacerbation was in 2018, making an acute exacerbation a less likely influencing factor to his AKI on CKD. Most recent sputum culture prior to admission grew *Pseudomonas aeruginosa.* Per chart review, patient had a baseline creatinine of 1.5–1.6 mg/dL with a GFR of 44–45 mL/min from previous labs. Decline in renal function occurred two years prior to presentation due to significant dehydration; he previously had a creatinine around 1 mg/dL. At that time, the patient was advised to increase fluid intake and follow with nephrology; he never attended this appointment.

After a minimal improvement in renal function with traditional IV fluids, elevated tobramycin serum levels revealed an inability to adequately clear the aminoglycoside, leading to kidney injury. Once the nebulized tobramycin was discontinued, the renal function slowly improved. Within cystic fibrosis literature, recurrent systemic use of aminoglycosides is commonly associated with long term renal damage and worsening creatinine clearance. The systemic absorption of nebulized tobramycin can be mildly detectable but not high enough to cause changes in creatinine or BUN when administered via a nebulizer [15,16,17]. Limited studies exist documenting the systemic effects that nebulized tobramycin can have on the renal system. A 2010 placebo-controlled, double-blind, randomized study of 300 mg nebulized tobramycin dosing found the typical serum levels 30 to 60 min after drug administration ranged from 0.54 μg/mL to 2.64 μg/mL (with a lower limit of detection at 0.18 μg/mL) [18]. A study comparing median serum levels following a single 600 mg once daily dosing of nebulized tobramycin, instead of the standard 300 mg twice daily dosing, were 3.44 μg/mL and 2.84 μg/mL with the AKITA and PARI-LC nebulizer machines, respectively. These serum levels were obtained immediately after administration (0.44 and 0.40 h) [19]. Our case presents a unique sustained elevation in tobramycin serum levels that are higher than previously published levels.

Advancement in research and medications has increased the life expectancy within patients with cystic fibrosis. As patients live longer, the risk for secondary complications to the disease such as AKI or CKD may increase in documented frequency. Presentations such as the one described in this case may become more common and may require clinicians to be more clinically aware of potential adverse side effects to these new novel agents within older cystic fibrosis patients such as our patient. Several case reports document systemic side effects from nebulized tobramycin. In a case from 2002, a 20-year-old patient with CF, receiving TIS for *P. aeruginosa*, developed an AKI; serum creatinine was 9.0 mg/dL (baseline of 0.6 mg/dL) with concomitant serum tobramycin level of 2.8 μg/mL. Acute tubular necrosis (ATN) was confirmed by renal biopsy; renal function was restored (1.6 mg/dL) after TIS was held [20]. This episode is the most recent documented case of renal damage related to nebulized tobramycin in a cystic fibrosis patient. Only one other case has been reported in the past eighteen years on this rare side effect within this population group. Including our case of AKI, there have been four other reported cases of kidney injury related to TIS: one in a patient with CF and three others (post heart transplant, complicated pneumonia, and COPD). In one report, a heart transplant patient receiving TIS via positive pressure ventilation for pneumonia had tobramycin serum levels of >2.0 μg/mL. The elevated level was postulated to be due to recent transplant graft failure complicated by cardiac and renal failure [21]. Our case had limited predisposing conditions despite baseline CKD (creatinine baseline of 1.5–1.6 mg/dL) and our patient received no immunosuppressant therapy. In another report, a woman undergoing imipenem-cilastatin, vancomycin, and inhaled nebulized tobramycin antibiotic treatment for sepsis secondary to pneumonia was reported to develop nephrotoxicity and worsening serum creatinine greater than her baseline of 2 mg/dL. Tobramycin trough levels were found to be 0.7 μg/mL with a worsening serum creatinine of 4.5 mg/dL. The patient ultimately required hemodialysis without return of renal function after resolution of her pneumonia [22]. The nephrotoxic effects reported in this case may have been the result of the combination of vancomycin and imipenem, and not TIS. The third case described a 73-year-old woman with COPD and newly acquired positive sputum culture for *Pseudomonas aeruginosa*. After four days of therapy, GFR decreased to 50mL/min and tobramycin level was 5.6 µg/mL; dialysis was initiated [23].

In regard to our patient’s progressive vestibular dysfunction (mild balance problems, hearing reduction, and loss of visual focus during movement), it was first thought to be related to a prior episode of neuronitis diagnosed one year ago. However, due to his acute worsening of symptoms five days before admission, it was concluded that our patient’s symptoms were most likely in response to elevated tobramycin levels. Similar acute vestibular side effects from TIS have been reported in prior case reports, with most formal research focusing on IV aminoglycosides effects [14]. Limited research has been performed documenting the prevalence of vestibular dysfunction in TIS. In a case report from 2004, it was documented that a female with chronic renal failure (creatinine 9.2 mg/dL) on hemodialysis with a chronic *Pseudomonas aeruginosa* infection who was receiving TIS was reported to develop reversible vestibular toxicity with associated dizziness, ataxia, and oscillopsia. Her documented tobramycin level was 19.2 μg/mL, which trended down to 9.8 μg/mL two days later withholding of TIS [24]. Our case presents similar findings as this report, and it helps to suggest our patient’s vestibular symptoms were connected to his AKI and elevated tobramycin levels. These findings go against previously supported conclusions that nebulized tobramycin does not cause ototoxic or vestibular injury [25]. The presence of new or worsening vestibular symptoms in the setting of TIS usage can be potentially used as a screening measure to assess for medication toxicity.

The information presented in our case documents important and relevant information on potential rare side effects for nebulized tobramycin that have only been anecdotally reported previously. Our case presents a unique account of tobramycin toxicity in a patient who is not acutely ill and has tolerated TIS for over two years without previous systemic side effects. Based on the WHO-UMC and Naranjo Probability Scale, our reported adverse drug reaction (ADR) receives a score of “9” with an associated causality as “Definite” [26]. This scoring suggests our ADR (elevation in creatinine) has a plausible time relationship with the nebulization of tobramycin. Additionally, reported withdrawal of the medication showed a plausible improvement in serum creatinine levels, concluding this event was a definitive pharmacologic phenomenon. These findings are critical to report to help aid clinicians when evaluating and treating similarly presenting patients with cystic fibrosis.

## 4. Conclusions

This case illustrates the potential for TIS to cause acute kidney injury and raises clinical awareness of the importance of monitoring nebulized antibiotic treatments in the face of advancing age and potential renal toxicity in a patient with baseline CKD. This case supports the benefit of obtaining tobramycin serum levels when evaluating patients on chronic daily suppressive nebulized tobramycin treatments, especially when presenting with acute or chronic renal dysfunction, elevated creatinine, and reduced GFR. Adjustments to daily dosing, regular monitoring of tobramycin serum levels, or discontinuation of treatment should be made to prevent permanent renal damage.

## Figures and Tables

**Table 1:** Laboratory Values. The table shows pertinent laboratory value trends one-year preadmission, during hospitalization (day 0 to day 9), at discharge (day 11), and after follow-up (day 41) for our patient.

Laboratory Test	Normal Range	Range One Year Preadmission	Day 0	Day 3	Day 4	Day 7	Day 9	Day 11	Day 41
Creatinine (mg/dL)	0.62–1.27	1.5–1.6	2.50	3.06	3.27	3.74	4.08	3.86	2.1
GFR ^1^ (ml/min)	>60	44–45	27	22	20	17	15	16	33
Potassium (mmol/L)	3.5–5.5	4–5	5.7	5.2	4.8	4.8	4.4	4.8	5.4
Hemoglobin (g/dL)	13.4–17.5	8–10	8.2	8.7	8.5	8.6	8.6	8.5	
Tobramycin Level after TIS ^2^ with Timing (μg/mL)				4.2 (2.5 h) 3.6 (8 h)	1.1 (24 h)				
Urinalysis			Normal			Normal			
Other		Respiratory Culture: 1 + Rare *Pseudomonas aeruginosa*	FENa ^3^ 3.8% (intrinsic), FEUrea ^4^ 59.3% (intrinsic), Bicarbonate 17 mEq/L			UNa ^5^ 72 mM, UOsm ^6^ 277 mOsm/kg, SOsm ^7^ 308 mOsm/kg H_2_O, UpH ^8^ 5			

^1^ GFR = glomerular filtration rate, ^2^ TIS = tobramycin inhalation solution, ^3^ FENa = fractional excretion sodium, ^4^ FEUrea = fractional excretion urea, ^5^ UNa = urine sodium, ^6^ UOsm = urine osmolality, ^7^ SOsm = serum osmolality, ^8^ UpH = urine pH.

## Data Availability

The data presented in this study is available on request from the corresponding author and approval by the consented patient involved in this report. The data is not publicly available to protect patient privacy and identity.
